# My studies of primates: Sex, affinity, and competition

**DOI:** 10.1007/s10329-023-01063-y

**Published:** 2023-04-04

**Authors:** Yukio Takahata

**Affiliations:** School of Policy Studies, Kwansei Gakuin University, Gakuen, Sanda, 669-1330 Japan

**Keywords:** Japanese macaque, Chimpanzee, Ringtailed lemur, Sexual behavior, Reproduction, Social relationships

## Abstract

In this essay, I summarize my research career, with reference to the early days of the Laboratory of Physical Anthropology (LPA) at Kyoto University led by Kinji Imanishi and Junichiro Itani. When I started conducting research on the sexual behavior of Japanese macaques in 1975, I made some unexpected observations. High-ranking males did not obtain high mating success. Estrous females often rejected the courtships of high-ranking males and chose to mate with lower-ranking males. Some male–female dyads exhibited long-lasting affinitive relations, but they avoided mating. Females frequently showed ‘excessive’ sexuality. Clear explanations for some of these observations do not exist. After that, I changed my study subjects several times from chimpanzees, Yakushima macaques (a subspecies of Japanese macaque), and ringtailed lemurs. It is difficult to summarize my findings into a consistent story. Instead, I review my research and experiences. Throughout my career, I kept two things in mind. The first was established by Imanishi at the Laboratory of Physical Anthropology at Kyoto University: to explore the evolution of human society. Second, I tried to understand seemingly incomprehensible phenomena using evolutionary theory. Despite adhering to these foundational concepts, things did not always work out as planned.

## Introduction

“Rather than trying to make an important discovery, it should be important to try to make your discoveries important.” (Shirakami 1972)

This paper summarizes my research career, with reference to the early days of Japanese primatology. This is neither easy nor straightforward. When I started conducting research in 1975, paradigmatic changes were in progress. Due to the rise of sociobiology and behavioral ecology (e.g., Wilson 1975), the interpretations of nonhuman primates and human behaviors were changing drastically. In addition, new standards for conducting observations were being developed (Altmann 1974). As all of this was taking place, I changed my research subjects and topics several times. I often left many questions unanswered without pursuing them further. In each case, I adopted an inductive rather than deductive approach. I searched for topics to study at my field sites instead of using a theory to develop hypotheses to investigate. In the 1990s, Alison Jolly told me that she had the impression that this mode of operation was one of the characteristics of Japanese primatologists. As a consequence, my tale might read like a series of unrelated and disconnected studies. I hope readers will try to understand them with an open mind and heart.

In 1972, I entered the Faculty of Science, Kyoto University, Japan. I selected my favorite courses during the third year. I was fascinated by the lectures on physical anthropology and wanted to join the Laboratory of Physical Anthropology (LPA). Three Japanese books influenced me the most as I was starting out. *Dobutsu no shakai* (*Animal societies*) published by Kinji Imanishi in 1972 taught me that each animal species forms its own type of society, which he called “specia.” *Reichorui no shakaikozo* (*Social structure of primates*) written by Junichiro Itani in 1972 described the main patterns of primate social structure and pointed out that they can be related to their phylogenetic relationships. Another book, *Hikaku Seitaigaku Zohoban* (Comparative ecology, enlarged edition), was written by Yoshiaki Ito (1966). Ito was an insect ecologist who later introduced sociobiology and behavioral ecology to Japan. In his book, Ito explained the basic concepts of animal ecology and pointed out two primary trends in animal societies: prolific and non-offspring-caring lifestyles and non-prolific and offspring-caring ones. He related these trends to life history theory. Apart from these three books, the lectures by Kenichi Shirakami also left an impression on me. He was a Professor of Embryology at Kyoto University and studied the early development of toads. The opening epigraph is taken from his book (Shirakami 1972). He taught us that theory is required to make our discoveries meaningful.

## Early days of Japanese primatology

### The start of Japanese primatology

In November 1948, Imanishi was an unpaid lecturer at Kyoto University. He was engaged in sociological research on semi-wild horses at Toimisaki Peninsula, Miyazaki, Japan, with two students, Shunzo Kawamura and Junichiro Itani. One day, Kawamura and Itani encountered a wild group of Japanese macaques and became interested in them. On December 3rd, they attempted to conduct a preliminary survey of the monkeys on Koshima Islet, 12 km north of Toimisaki Peninsula. Although they found only food remains and feces, Itani (1985a) was to note later that Japanese primatology started that day.

In 1951, the “Primate Research Group” was formed in the Laboratory of Animal Ecology at Kyoto University under the supervision of Denzaburo Miyadi, a Professor of Animal Ecology. Miyadi studied aquatic organisms inhabiting lakes and rivers in Japan, and from 1964 to 1976, he was the director of the Japan Monkey Center (JMC). The group’s goal was to clarify the social structure and evolutionary history of primates, and to understand the origin of human society (Imanishi 1972). Early investigations made slow progress due to the monkeys’ wariness of humans. Research accelerated after they succeeded in habituating monkeys through provisioning at Koshima (Kawai 1965) and Takasakiyama (Itani 1954) in 1952. Following this, many groups were provisioned mainly for tourism (e.g., Boso, Shodoshima). Masao Kawai (1965) reported sweet potato washing and wheat placer mining as two newly acquired pre-cultural behaviors. Kawamura (1958) documented two principles underlying dominance rank relationships among females: (1) the daughters rank below their mother, and (2) the younger sister is ranked higher than the older sister. The second observation became known as the principle of youngest ascendency. Imanishi (1957, 1960) summarized these results and highlighted the signs of “cultures” among nonhuman primates. He proposed “identification” as a system for transmitting such non-innate behaviors to the next generations. Although their discoveries depended on provisioning, artificial feeding biases some aspects of monkey life (Sugiyama 2022). For instance, social changes occurred due to increased levels of aggression and demographic changes, including increases in group size and skewed sex ratios (Hill 1999).

In 1956, the JMC was established at Inuyama, Aichi, with the financial support of the Nagoya Railroad Co., Ltd. for primatological research, monkey supply, and education. Imanishi became the director, while Itani and Kawai worked as research staff members. In 1957, JMC started to publish this scientific journal, *Primates*. In 1958, another organization was formed. Osaka University provisioned a Japanese macaque group at Katsuyama, Japan (Nakamichi 2021). Naosuke Itoigawa led this project (Itoigawa et al. 1992), and many primatologists have been trained by him (e.g., Nakamichi and Shizawa 2003, Yamada and Nakamichi 2006).

In the late 1950s and early 1960s, overseas primate research began. Yukimaru Sugiyama (1965) conducted a ground-breaking study of infanticide by Hanuman langurs in India (Sugiyama 2022). In 1958, Imanishi and Itani conducted a field survey of wild gorillas in Africa. When revolution broke out in the Republic of the Congo (Léopoldville) in 1960, Itani switched to chimpanzees along the east coast of Lake Tanganyika in Tanzania. A large team including cultural anthropologists and paleoanthropologists formed the Kyoto University African Ape Expedition (KUAPE). In 1961, a base camp was set up at Kabogo, Tanzania, to study chimpanzees, but the habituation process was unsuccessful. In 1963, Itani moved the base camp to Kasakati inland from the lakeside.

### Establishment of the laboratory of physical anthropology, Kyoto University (LPA)

In 1962, the LPA was established. As its professor, Imanishi envisioned studying the evolution of human society from multiple perspectives. The associate professors were Jiro Ikeda, a physical anthropologist (Ikeda and Hayama 1982) and Itani. Their assistant professors were Sugio Hayama, an anatomist (Hayama 1970) and Sugiyama. After Imanishi retired in 1965, Itani devoted himself to studying wild chimpanzees, but it was challenging work. Jane Goodall (1965) had already succeeded in provisioning chimpanzees at Gombe, Tanzania. She had concluded that there were no stable groups other than a mother and her offspring.

On September 3rd, 1965, Itani and Akira Suzuki (1967) made an important discovery. They observed 43 chimpanzees moving in a row in a woodland area called Filabanga. They speculated that a large group consisting of 30–50 individuals was the social unit within which members fissioned and fused. Itani assigned three students to different field sites. Kosei Izawa (1970) continued research at Kasakati. Takayoshi Kano (1971) attempted to habituate chimpanzees at Filabanga. Toshisada Nishida (1968) started to study chimpanzees at Mahale along the shore of Lake Tanganyika.

In 1966, Nishida succeeded in provisioning chimpanzees at Mahale. He confirmed that chimpanzees formed multi-male and multi-female groups in which individuals constantly split apart and came together. He called such social units “unit groups,” synonymous with the term “communities” used at Gombe and other field sites. In 1966, Sugiyama conducted research in the Budongo Forest, Uganda, and also pointed out that wild chimpanzees lived in fission–fusion groups (Sugiyama 1968).

In the 1980s, Kano (1992) succeeded in provisioning wild bonobos at Wamba, Zaire, where many Japanese primatologists were subsequently trained (e.g., Idani 1991, Ihobe 1992, Hashimoto 1997, Furuichi 2011). Izawa started to study platyrrhine primates (Izawa 1975) and wild, non-provisioned Japanese macaques on Kinkazan Islet, Miyagi, Japan. Many important findings have come from these field sites (e.g., Nakagawa 1989, Nishimura 2003, Shimooka 2005). In 1967, the Primate Research Institute (PRI) of Kyoto University was established in Inuyama, Japan. Many primatologists, including Kawamura, Kawai, and Sugiyama moved there. Additionally, the PRI started to train graduate students.

### Activities of LPA during the 1970s

After his fieldwork at Filabanga, Itani’s research interests focused on two topics: the evolution of nonhuman primate social structure and ecological anthropology of African people. The latter reflected the fact that Itani and some of his students were becoming increasingly interested in the natural and cultural features of Africa. When I joined the LPA in 1976, four teachers were there. Ikeda led the laboratory as its professor. Itani was associate professor. Hidemi Ishida, a physical anthropologist (Ishida and Pickford 1997), and Reizo Harako, an ecological anthropologist (Harako 1976) were assistant professors.

Each student chose a topic to study from three areas: anatomy, humans, or primates. Studies that emerged involved non-metrical cranial variants of modern Japanese people (Mouri 1976), hunter-gatherers (Ichikawa 1981), and agricultural people (Kakeya 1987). For primates, ethological research was featured. Akio Mori (1977) analyzed interactions between Japanese macaques and found that, except for mothers and their offspring, interactions did not occur frequently. When unrelated monkeys approached others, behavioral/vocal signals (= greetings) were uttered. Tomoo Enomoto (1974) classified the sexual behaviors of Japanese macaques and found no relationship between male rank and copulation frequency. I used these results later to develop my own research on the social relations and sexual behavior between male and female Japanese macaques in Arashiyama (Takahata 1982a, 1982b). In overseas studies, Suehisa Kuroda (1980) and Koji Kitamura (1983) reported that bonobos showed high levels of tolerance toward each other within and between groups and that their society might be integrated by male/female affinity. Juichi Yamagiwa (1983) began to observe wild gorillas.

At seminars, Itani listened carefully and patiently to the reports of his students. Based on those discussions, Itani wrote reviews about the evolution of primate societies (Itani 1977, 1985b). His phylogenetic diagram of social structures might be the primate version of the societies of eusocial insects (e.g., bees and ants). However, his efforts were limited to classification and phylogenetic arrangements, and his analysis of evolutionary factors did not fully reflect the paradigmatic changes taking place in evolutionary biology at the time. I believe that considerable progress would have been made by combining the phylogenetic study of Itani and the life history theory of Ito (1966). However, their students, including myself, failed to merge these disciplines.

## Arashiyama macaques

### The start of my research

In October 1975, I was a fourth-year student at Kyoto University. Itani suggested that I write a thesis based on a study at Iwatayama Monkey Park, Arashiyama, Kyoto. I hesitated because criticism of studies of provisioned monkeys was increasing (see Sugiyama 2022), but I reconsidered and thought that it was good idea to gain field experience at Arashiyama before entering graduate study at the LPA. Itani proposed sex, play, and grooming as possible topics. I chose sex, and Itani gave me papers written by Carpenter (1942) and Enomoto (1974) to read.

At Arashiyama, a group of Japanese macaques (*Macaca fuscata fuscata*) was successfully provisioned for tourism in 1954. Because these monkeys were fed for a long time, their group size had become huge. In 1966, the original group fissioned into Arashiyama A and B groups. The former group was formed by the females of the 1st to 7th-ranking kin-groups, and the latter group consisted of the females from the 8th to 16th ranking kin-groups. Subsequent studies have revealed that Japanese macaques exhibit female philopatry (e.g., Koyama 1970). Males usually leave their natal group around sexual maturity, while females remain and form kin-groups. In 1972, Arashiyama A group was captured, sent to Texas, USA, and renamed the “Arashiyama West group” (Fedigan and Asquith 1991). Arashiyama B group continued to be provisioned at the park and contained about 210 monkeys in October 1975. The socioeconomic sex ratio (SSR) was low, at 0.261. Each year, 40–50 infants were born, and the group size increased to about 270 monkeys in September 1977. Baldev Singh Grewal (1980) was studying social relationships as a graduate student at PRI and kindly taught me how to identify individuals in the group.

I soon made some unexpected observations. Females frequently rejected courtships from high-ranking males. In addition, some females had affinitive relations with specific males. When such females came into estrus, these male–female dyads avoided mating. Their sexuality, or lack of it, could not be understood without knowing the nonsexual relationships they shared. I planned to observe them until at least 1978, and Itani approved my plan. My observation methods were in accordance with the methods of Altmann (1974) and the studies of small mammals by Takeo Kawamichi (Kawamichi and Kawamichi 1979). Kawamichi had studied the social relationships of pikas, tree shrews, squirrels, and giant flying squirrels since the 1960s. Based on these experiences, I later published a booklet in Japanese for students on how to conduct research on Japanese macaques. In it, I covered how to make a research plan, select a field site, and conduct observations (Takahata 1985a).

In 1976, Nobuo Asaba became the new owner and director of the monkey park, and he was in search of the best way to manage it. The traditional monkey park was an amusement park and gradually declining in popularity. There was a need for a new kind of park management. I taught him how to identify individual monkeys. While observing monkeys at the feeding ground, I also witnessed interactions between human visitors and monkeys. The most memorable visitor was a teenage boy suffering from intellectual disabilities. In the 1970s, the visitors were allowed to feed monkeys (e.g., peanuts) freely outside the park office building, but this was later, banned by Asaba. The boy appeared once every few months, and gleefully gave a lot of food to the monkeys. It was a happy time for him, as he smiled. Perhaps this served as form of animal therapy.

### Japanese macaques: Three research topics

**Fig. 1:**
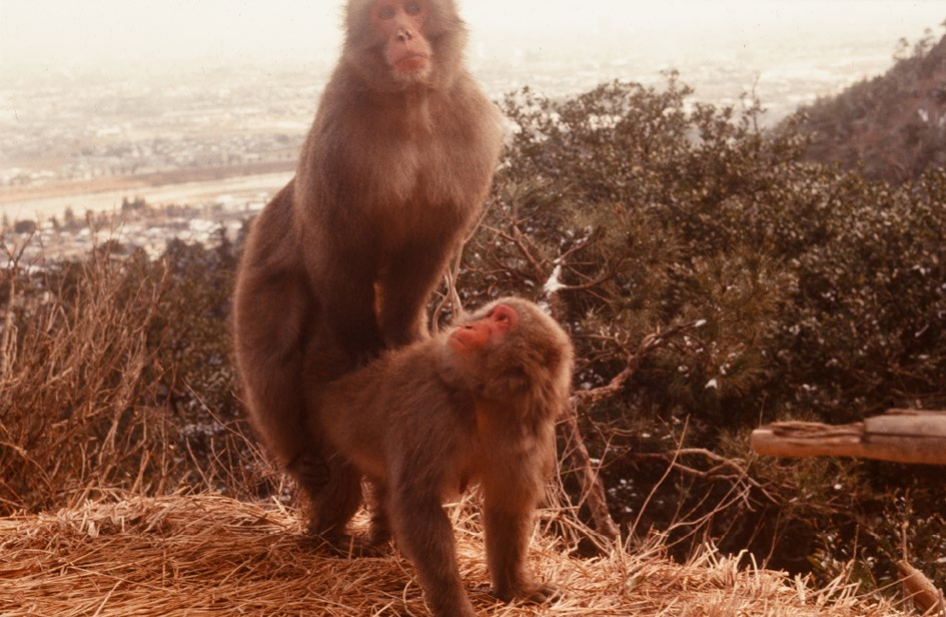
The alpha male (M-59♂) copulating with a high-ranking female (Ch-67♀)

I investigated three main research topics in my studies of Japanese macaques: reproductive biology, male–female nonsexual relationships, and socio-sexual behaviors. For the first, I recorded the signs of estrus daily, vaginal bleeding, semen plugs, and copulations (Fig. 1) of all adult and adolescent females during the 1976–77 mating season (Takahata 1980). The mean length of the menstrual cycle was 26.5 days, and females usually entered estrus around the middle of their cycle, near the time of ovulation. However, a considerable number of females showed irregular estrous cycles, including peri-menstrual or prolonged estrus lasting > 30 days. In addition, 86% of the 94 conceptions occurred in the first half of the mating season, meaning that most of the copulations in the latter half of the mating season were not linked to pregnancy. This is partly because pregnant females exhibited post-conception estrus, potentially confusing males about paternity. In any case, their estrus was not always limited to the period around ovulation. A similar phenomenon of ‘excessive’ estrus was also reported in a non-provisioned, wild group of macaques on the island of Yakushima (Okayasu 1992). It was not found, however, in another non-provisioned group on Kinkazan Island (Fujita et al. 2004). At Kinkazan, a cooler more temperate environment may have led pregnant females to focus on feeding rather than maintaining prolonged estrus.

Excessive estrus might be related to the evolution of “concealed ovulation” or the disappearance of estrus in human females. Primatologists, including myself, have noted how close monkey sex is to that of humans, emphasizing that estrus has diverged from ovulation (Hrdy and Whitten 1987). In addition, human sexologists sometimes argue “how close human sex is to that of nonhuman animals” (e.g., Udry and Morris 1968). The subtle differences between human menstrual and primate estrous cycles may provide insights into this issue.

On the other hand, ‘efficient’ pregnancy was revealed in my work, which may be necessary for seasonally breeding primates, such as Japanese macaques. Three-quarters of all pregnant females conceived in their first estrous cycle (Takahata 1980). Similarly, all females were impregnated in the first ovulation in an enclosed social group at PRI (Mitsunaga et al. 1992) and a non-provisioned group at Kinkazan (Fujita et al. 2004). Thus, female Japanese macaques might become pregnant in a much shorter time than female olive baboons, who become pregnant only after 4–6 estrus cycles (Smuts and Nicolson 1989), and female chimpanzees, who become pregnant on average during their sixth estrous period after post-partum resumption of sexual activity (Takahata et al. 1996).

**Fig. 2:**
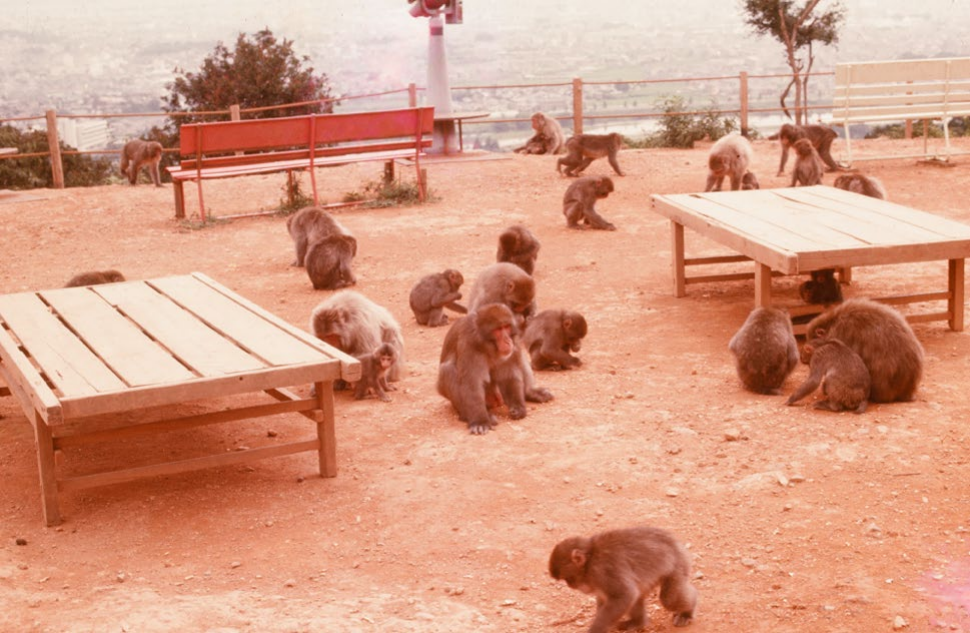
Alpha male, M-59♂ feeds in the center of the photo. The whitish female on his left is Op-60♀, who had been in a PPR with him for more than 10 years

I analyzed adult male–female relations based on data obtained during three non-mating seasons (Takahata 1982a). As Mori (1977) pointed out, most male–female dyads kept their distance from each other without ever grooming. However, some unrelated males and females maintained remarkable and long-lasting spatial proximity to each other and frequently groomed, suggesting the existence of some kind of affinitive relationship between them. Such an association had been called a ‘peculiar-proximate relation’ (PPR)” by Kitamura (1977) among male–female pairs at Takasakiyama. In 1976, at Arashiyama I identified 61 PPR dyads based on frequencies of proximity within 3 m and grooming (Fig. 2). Females obtained beneficial ‘proximity effects’ from their PPR males. Low-ranking females could dominate higher-ranking ones when near their PPR males. In return, the males acquired female followers, but not mating partners. During my study, most PPRs lasted two or more years. Recently, Suzuki et al. (2023) analyzed the process of fission of the Arashiyama B group that occurred in 1986. They found that several past PPRs between females with a former alpha male might have influenced their choice of which group they ended up belonging to.

PPRs seemed to be formed through two processes. First, after a long-lasting consortship, the dyad continued an affinitive relationship in the following non-mating season. Second, daughters formed PPRs with the males who had been in PPRs with their mothers. From 1975 to 1976, an adult male was estimated to have formed PPRs with 1.44 ± 0.73 females per mating season (Takahata 1982a). Once PPRs were formed, the pairs began to avoid mating; “sex and friendship” were not always associated; differing from olive baboons described by Smuts (1985). Some PPR pairs might experience sexual aversion due to a process similar to the “Westermarck effect” (Westermarck 1891), especially the daughters of females with close social relationships with adult males. For males, the acquisition of PPRs was not directly connected to increased mating success. Instead, the formation of PPRs might encourage high-ranking males to leave the group because they lead to a decrease the number of mating partners (Huffman 1992). As an indirect benefit, with more female followers high-ranking males may be able to maintain their ranks in rivalry with other adult males. I had few observations of agonistic interactions between males, however, to test this hypothesis. Anecdotally, when a solitary male (Sol-761♂) immigrated into the B group, he formed a PPR with a female (S-5969♀) through a consortship and secured a stable position within the group. This case may indicate that males can transfer smoothly to groups by forming PPRs with female members.

I conducted a study of socio-sexual behavior during the 1976–77 mating season (Takahata 1982b). Higher-ranking males frequently engaged in courtships with estrous females and interfered with the copulations of lower-ranking males, consistent with the priority of access model (Altmann 1962). However, estrous females often rejected the highest-ranking males' attempts to mate and instead consorted with lower-ranking males. Most of the pairs, who had their matings disrupted, quickly resumed copulating. Consequently, no relationship existed between adult male dominance rank and mating success. In the 1970s, DNA paternity tests did not yet exist; but they were later successfully applied to Japanese macaques in the late 1980s (Inoue et al. 1992). I determined the number of times females copulated with males when they were likely to conceive by backtracking from the dates of birth of their offspring. High-ranking adult males were involved only 15% of such copulations.

I concluded that female choice (Small 1993) is decisive for mating partner selection in the Arashiyama macaques. Later, Michael Huffman (1987, 1992) reported a similar finding. Is this common among Japanese macaques? Along with colleagues, I compared the relationships between adult male rank and mating success in several provisioned groups over 12 mating seasons (Takahata et al. 1999). We found a significant correlation in only one case. Why did dominant males fail to attain high mating success? There may be multiple reasons for this. Female physiology (e.g., simultaneous estrus, excessive sexuality) and female choice may promote promiscuity. PPR females may reduce the number of females that high-ranking males can mate. Finally, high-ranking males may adopt a ‘best female strategy’ focusing their mating efforts on a few rather than attempting to mate with multiple females in a ‘many female strategy.’

How many years do mating relationships last? Based on the data collected over seven mating seasons, Huffman and Takahata (2012) found that most mating pairs lasted only one to 2 years since copulation rates decreased after three or more years. Thus, males and females continually changed mating partners throughout their lives. This was consistent with the results of a paternity analysis conducted on a captive group (Inoue et al. 1992).

At Arashiyama, there was also no consistent relationship between female rank and reproductive success (Takahata 1980, Wolfe 1984, Koyama et al. 1992; Chalmers et al. 2012). This finding was similar to those on white-faced capuchin monkeys (Fedigan 2022) but different from those on geladas (Dunbar and Dunbar 1977) and olive baboons (Smuts and Nicolson 1989). By contrast, Itoigawa et al. (1992) reported that the fecundity rate of females increased as a function of matrilineal dominance rank at Katsuyama. In Koshima, some of the most dominant females also exhibited high reproductive success (Watanabe et al. 1992). The reason for this diversity in female fecundity among Japanese macaques remains unclear. Later, I obtained different results in wild ringtailed lemurs (Takahata et al. 2008). In medium and small groups, female rank did not appear to affect reproductive performance. By contrast, low-ranking females in large groups had fewer surviving infants than did mid-ranking females. Reproduction by subordinate females might have been suppressed by intense intra-group competition in large groups. These findings call for more studies on the relationship between female rank and reproductive success in female-bonded primates.

I also analyzed the avoidance of matrilineal inbreeding (Takahata 1982b). Males and females who were close kin (*r* = 1/2–1/8) rarely mated, but those who were distant kin (*r* < 1/8) mated as frequently as pairs who were unrelated. There was some inhibition of mating among distant kin dyads, however, because male ejaculation rates were low in these pairs. With my colleagues, I later analyzed data recorded during seven mating seasons and found that 2.9% of all copulations involved matrilineal inbreeding (Takahata et al 2002). In this study, close kin dyads strongly avoided mating, but remote kin dyads did not.

Do monkeys retain memories of their kin relationships? In Arashiyama, some adult sons remained in their natal groups after sexual maturity, which provided an opportunity to investigate this. In 1976, out of nine adult males in the group, two brothers (K-63♂ and K-65♂) were born in the Arashiyama B group and never left. It was difficult to deduce their kin relations from only social interactions, since both of them appeared to have less frequent contact with kin-related females but had PPRs with unrelated females. However, mating was rarely observed between members of these kin-related dyads, suggesting that they might have had some memories of early affinitive ties.

Atypical sexual behaviors also occurred. In the 1976–77 mating season, two-thirds of the adult and adolescent females engaged in homosexual behaviors (Takahata 1980, 1982b). This behavior might be related to the bias in the SSR. However, I hesitated to conclude that it was simply compensatory behavior by males. Female–female pairs were relatively long-lasting and more active and aggressive than other estrous females. They mounted each other endlessly. This differed from male–female copulations that ended with ejaculation. Additionally, homosexual behaviors rarely occurred among kin-related females. These observations made me think that female homosexual behavior was different from heterosexual behavior. Based on Carpenter’s (1942) suggestion, I checked whether there was a relationship between its occurrence and pregnancy. Most of the adult females who showed homosexual behaviors gave birth in the next birth season. They engaged in significantly more homosexual behavior after conceiving than before. By contrast, adolescent females who displayed homosexual behaviors infrequently gave birth in the next birth season, and no difference in the frequency of homosexual behavior was observed before and after conception. Regrettably, I do not have any further data to add to this discussion. Later, Leca et al. (2015) analyzed the long-term data on female homosexual behavior at Arashiyama. Their results did not support the "heterosexual deprivation hypothesis,’’ which suggests that female homosexual behavior is due to a shortage of male mates, or the ‘‘lack of opposite-sex sexual competitor hypothesis," which proposes that females have greater access to female mates when male sexual rivals are scarce. Instead, their findings were consistent the ‘‘bisexual preference hypothesis,’’ which holds that female homosexual behavior can be attributed to female preference for specific female mates compared to specific male mates.

Copulation-like mounts by immature males between the age of 1.5–3.5 years old frequently occurred (Takahata 1980). Immature males mounted estrous females in series but without ejaculation; ejaculations were observed when males reached 4.5 years of age, as reported at Takasakiyama (Nigi et al. 1980). Most of their partners were adolescent females but also included some adult females. Why did adult females respond to immature male copulations? I have no answer. Premenarchal females were indifferent to sexual activity, in contrast to immature males. In another case, when a 3-year-old female, Sh-6272♀, became estrus probably for the first time, she exhibited courtship behavior for several days to me. She masturbated by rubbing her vulva on my shoes. When I grabbed her back, she presented.

In sum, Japanese macaque sexuality is diverse. I remember a conversation with Linda Wolfe, who came to Japan in 1977 to compare the sexual activities between Arashiyama West and B groups. One day, we observed a copulation. At the last mount, the male ejaculated, and the female looked back at the male and called out. Wolfe asked me, “Do you think she feels orgasm?” I replied, “Of course! I think she feels orgasm.” Wolfe might have been thinking about the evolution of orgasm in human females.

In July 1978, I left Arashiyama. I had been worried about the problems posed by managing the monkeys, particularly those related to the rapid population increase. It seemed inevitable that the population required control through artificial manipulations, perhaps involving capture and culling, but there was no consensus about what to do. It was time for me to say goodbye to these monkeys.

### After Arashiyama: analysis of long-term data

It took me a long time to analyze the data I obtained at Arashiyama. Unlike today, there were no personal computers or software for word processing and statistical analysis. Additionally, we could not search for references online. I was the last LPA student to write a doctoral thesis on an IBM electric typewriter.

After leaving Arashiyama, I had several opportunities to analyze long-term data. In 1987, I reported the diachronic changes in female dominance ranks (Takahata 1991a) at a conference organized by Linda Fedigan and Pamela Asquith (1991). It was held to exchange opinions between the Japanese and Western primatologists who worked at Arashiyama East and West (Fedigan 2022). I compared the dominance ranks of adult females recorded in 1966 (Koyama 1970) and 1976. In 1976, most kin-related dyads followed Kawamura’s principles. No significant changes occurred in the rank order of female kin-groups from 1966, except for a few “upwardly mobile” females. Most of these females had PPRs, with high-ranking males. I also described some exceptional cases of severe aggression between kin-related females, in which the eldest daughters outranked their mothers for three generations. In 1966, Koyama (1970) observed that a female named Mino persistently attacked her mother, Kojiwa, and outranked her, becoming the alpha female of B group. Nine years later, I happened to see that Mi-63♀ (Mino's eldest daughter) outranked Mino without employing aggression. Immediately after that, Mi-6369♀, Mi-63♀'s eldest daughter, suddenly bit Mi-63♀, who became submissive to her. A half hour later, however, Mi-6369♀ was seen to be seriously injured and outranked by her mother. I did not see the attack on Mi-6369♀. Then Mi-63♀ repeatedly tried to groom the injured Mi-6369♀ as if to try to reconcile with her daughter, but Mi-6369♀ only ran away grimacing. In the winter of early 1979, Mi-6369♀ together with her daughter Mi-636974♀ attacked Mi-63♀, seriously injuring Mi-63♀. Later, Asaba found Mi-63♀'s body in the pool of the park after the ice melted in the early spring. She perhaps fell in during her weakened state while trying to drink or when passing by on the slope above (MA Huffman, personal communication). It seems that Mi-6369♀ may have been involved in killing her mother. These cases indicate the presence of intense competition among kin-related females for α-status. This conclusion is tentative, however, because the data were too fragmentary.

In the 1990s, I participated in the analysis of birth records recorded over 30 years (Koyama et al. 1992). The results of this study were published in *Primates* with similar reports from Koshima (Watanabe et al. 1992) and Katsuyama (Itoigawa et al. 1992). At Arashiyama, female rank had no apparent effect on female reproduction. Female birth rates had an inverted U-shaped curve relationship with age, and some old females displayed an apparent post-reproductive life span (PRLS). Takahata et al. (1995) compared the PRLS with those of Yakushima macaques and Mahale chimpanzees. For old females, PRLS was 4.5 years (16% of lifespan) at Arashiyama and 3.6 years at Yakushima. At Mahale, old female chimpanzees spent 6.6 years without giving birth before death, but PRLS was only 2.2 years (about 4–5% of lifespan) because female chimpanzees required 4.4 years before weaning their last offspring. By contrast, Pavelka and Fedigan (1991) failed to show the existence of PRLS in monkeys at Arashiyama West. Ichino et al. (2015) also found that PRLS did not exist in ringtailed lemurs at Berenty Reserve, Madagascar.

### A short stay in a tropical forest in Sumatra

After leaving Arashiyama, a documentary TV film company (Nippon A-V Productions) asked Professor Itani for a scientific adviser to film an orangutan rehabilitation center in Indonesia (for this company, see Ichioka 1988). I stayed at the Bohorok Orangutan Center, Sumatra, for about 50 days on my first overseas research trip. The center rescued immature orangutans captured by humans and returned them to the forest. I witnessed a flanged male emerge from the forest and mate with a rehabilitant female. Around the center, wild siamangs, lar gibbons, long-tailed macaques, and Thomas’s langurs coexisted. The observation conditions were very difficult, but the sympatric primates left a strong impression on me. I had been invited to join a research project organized by Toshisada Nishida at Mahale where I was to conduct research on sympatric monkeys. Another memorable scene was a rubber plantation next to the center. A group of Thomas's langurs inhabited the plantation. There, the Indonesian workers gathered latex into balls daily. They piled the balls up and waited for trucks to pick them up.

## Mahale chimpanzees

### Mahale and national park plans

I visited Mahale in July 1979 as part of Nishida’s project. My subjects for study were switched to chimpanzees (*Pan troglodytes schweinfurthii*) because other researchers changed their schedules. I conducted research at Mahale intermittently for a total of 3 years until September 1984.

At Mahale, the K and M chimpanzee groups were habituated. These groups had an antagonistic relationship. During the 1970s, K group was observed by Nishida and Shigeo Uehara (1982), and M group was observed by Kenji Kawanaka (1982) and Kohshi Norikoshi (1982). Mariko Hasegawa (Hiraiwa-Hasegawa et al. 1984), Toshikazu Hasegawa (Hasegawa and Hiraiwa-Hasegawa 1983), Hitoshige Hayaki (1985), and Hiroyuki Takasaki (1983) participated in chimpanzee research in the early 1980s. Nishida and M. and T. Hasegawa were the first Japanese primatologists to promote sociobiology and behavioral ecology. They played a significant role in the subsequent introduction of these disciplines to Japan. Other Japanese primatologists, including me, had a ‘fence sitter’ attitude about these fields of study at the time.

In the 1970s, Nishida and Itani promoted a plan to build a national park in Mahale with the aid of the Japan International Cooperation Agency (JICA). The Mahale Mountains Wildlife Research Centre (MMWRC) was established, and Tanzanian staff helped us. Ramadhani Nyundo and Mohamedi Seifu Kalunde, who worked on Nishida’s early research and sometimes coauthored our papers, were especially important collaborators. The Mahale Mountains National Park was gazetted in 1985. The park covers an area of 1613 km^2^ and is located about 128 km south of Kigoma. Nishida continued his research there until he died in 2011. Currently, Michio Nakamura and his colleagues are continuing the long-term research there (Nakamura et al. 2015).

**Fig. 3:**
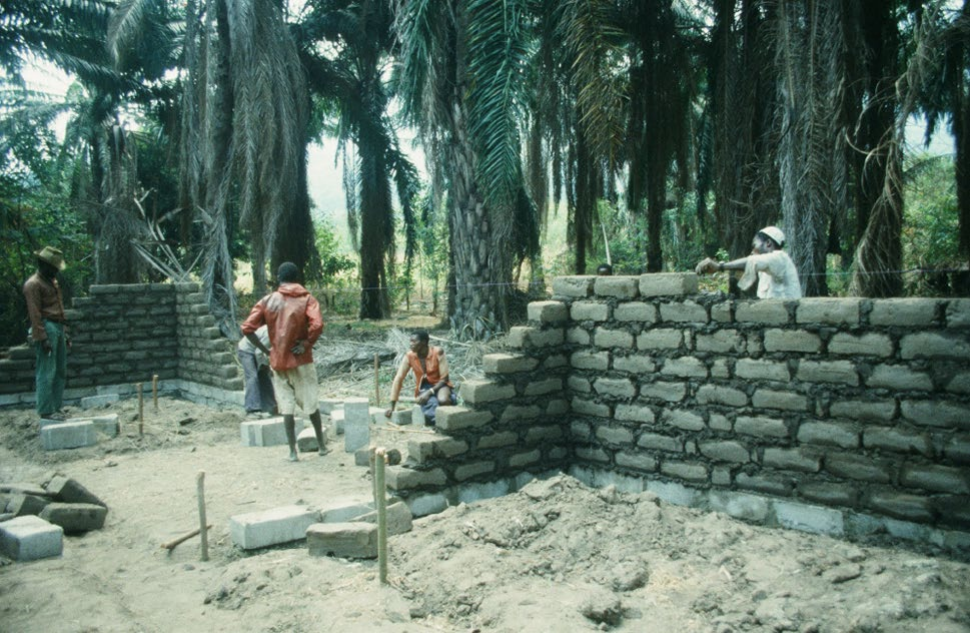
Guest house at Mahale under construction during my tenure as a JICA expert

From 1982 to 1984, I was the JICA expert in charge of managing MMWRC (Fig. 3). During my term, Tanzania was in the final stages of its socialist government, and its economy was in ruin. Petrol and other materials used to maintain MMWRC were difficult to acquire. Traveling was extremely time consuming. Few people visited Mahale. From September 1982 to February 1983, William McGrew and Anthony Collins investigated termite fauna and tool-using behavior by chimpanzees (Collins and McGrew 1987). At Christmas that year, a group of visitors, including David Bygott and Jeannette Hanby, visited Mahale. In 1984, just before I left Mahale, Richard Byrne arrived with his wife to conduct research.

During my stay, we followed chimpanzees through the forest, and the amount of sugar cane given to them was minimal. I believe that it may be difficult for wild chimpanzees to spontaneously mimic human behaviors. We stored the sugar cane in a galvanized iron hut. Its door was not locked, and the door latch could be opened in three steps. However, the chimpanzees did not understand what I was doing when opening the door in front of them. They only hit the door with their hands, and the door would not open because it sank into its frame.

Each day I searched for the chimpanzees with Tanzanian staff members. When we could not find chimpanzees, we talked in Swahili, asking each other questions. For example, “How different are Japan and Mahale?” To their surprise, it rains every month in Japan, something quite different from Mahale, where the year is clearly divided into rainy and dry seasons. Once, Ramadhani questioned me about the BBC Swahili news broadcast. “Kwa nini Mao ameacha kijiji” (Why has Mao abandoned the people’s commune?). It took me awhile to realize that by Mao, he meant the former Chinese communist leader Mao Zedong (actually, Mao had already died several years ago). I felt his anger regarding the compulsory villagization (Ujamaa village) policy by the Tanzanian government, which had adopted socialist policies, just like the People’s Republic of China. Nevertheless, Ramadhani learned that the Chinese government had abandoned the people’s communes. After I left Mahale, Tanzania underwent a transformation from a socialist economy to a market economy in 1985. Along with this, the compulsory villagization policy was also abandoned.

### Social relations among male chimpanzees

In the 1980s, M group was a large group of 86–101 individuals (Nishida et al. 1990). I had planned to investigate the relationships between adult males and females, just as I did at Arashiyama with the macaques. I could not carry out this plan, however, because many females were not yet fully habituated to observers. More importantly, the most significant social partners for prime males were other prime males. They maintained close social ties with each other, called ‘male bonds.’ These bonds were the core of their patrilineal society.

In 1981, I recorded social relationships among the alpha male, five prime males, and four young adult males (Takahata 1990a). At first, it was difficult to establish their rank order, except for the alpha male, Ntologi. Agonistic interactions occurred infrequently, and pant grunts/barks were usually uttered by the subordinates to Ntologi. It took considerable time before I could determine the rank order among the other males. All of my observations indicated the prominent role which Ntologi played and that he was the focus of attention. Other males unilaterally approached and maintained proximity to him. When low-ranking males approached Ntologi, they uttered pant-grunt/barks, confirming their subordinate status to him. Social interactions between Ntologi and other prime adult males differed than those between him and young adult males. Ntologi actively returned the grooming of prime males, but he had no grooming interactions with young adult males. When Ntologi moved first, prime males frequently followed him, but young adult males did not. In hunting episodes, Ntologi often shared meat with other prime males. Nishida et al. (1992) proposed that Ntologi used meat-sharing as a coalition strategy.

Among the other prime males, the dominants and subordinates bilaterally approached, followed, and groomed each other. They infrequently uttered pant grunts/barks and acted as equals. In contrast, there was an insurmountable gap between prime and young adult males. Prime males were unilaterally approached, followed, and groomed by young adult males. Young adult males seemed to fear older males, but also might be attracted to them because they have to establish their positions in the male bonded group of prime adult males in the future. Among young adult males, the subordinates tended to approach and follow the dominants, and the subordinates sometimes uttered pant grunts/barks. However, such behaviors were infrequent, and they did not always maintain close proximity to each other. The lack of frequent interactions between young adult males gave me a different impression from young Japanese macaque males, who often formed peer groups. The age-related changes in male social relationships reminded me of the life stages of humans.

I also analyzed the relationships between adult males and females (Takahata 1990b). Prime male chimpanzees dominated adult females. It is quite different from bonobos, in which male–female dominance is equal or equivocal, and females are dominant over males during feeding (Furuichi 2011).

Since chimpanzees display male philopatry, the relationships between mature males and their mothers deserves attention. In the early 1980s, three mother–mature son pairs existed in M group. But the two mothers were too shy, and I could observe only one pair in detail. They frequently interacted when the mother was not cycling. It was quite different from the kin-related adult male and female dyads of Japanese macaques in Arashiyama, where kin-related pairs did not maintain spatial proximity or interact socially with each other frequently. In both chimpanzees and Japanese macaques, adult sons were not present when their mothers were in estrus, and no mating behavior was observed.

For unrelated females, female reproductive state influenced their relationships with adult males. Non-cycling females unilaterally approached and greeted males, but tended to walk away without further interactions with males. The alpha male showed possessive mating patterns around their estimated time of ovulation near the period of maximum sexual swelling (Hasegawa and Hiraiwa-Hasegawa 1983). In contrast, other males mated females opportunistically (*ibid*.).

### Group extinction, adult female transfer, and infanticide

K group went extinct during my time at Mahale. The dissolution of K group was a significant loss for the Mahale chimpanzee study because it precluded the opportunity to investigate intergroup relationships between members of two habituated groups.

In 1967, K group contained 29 chimpanzees, and no one would have predicted that it would dissolve. Initially, adult males disappeared one by one from 1969 to 1975. Nishida et al. (1985) surmised that several K group males were killed by the M group males, as reported at Gombe (Goodall 1986). I speculate that several factors played a role in the dissolution of K group (Takahata 2015). K group failed to recruit adult males because few male infants survived to adulthood in the 1960s and 1970s. As noted above some males may have been killed by members of M group. In addition, several males died of old age, and an adolescent male named Masisa died from apparent psychological stress, as described below. Thus, there was high mortality in K group adult males between 1975 and 1979, and M group males outnumbered those in K group.

Females may have also contributed K group’s extinction. First, few females immigrated into K group as its numbers declined. Second, K group cycling females started to immigrate into M group or some other unknown groups to obtain mates or to avoid aggression from neighboring groups. After only one old male named Kamemanfu remained in 1979, many K group females transferred to M group. Lactating females stayed, but they eventually left K group when their infants were weaned and they resumed cycling. Estrus might be considered a passport for transferring into a new group.

Young males born in K group had a difficult time. Two juvenile males transferred into M group with their mothers (Takahata and Takahata 1989). It is unclear why M group males did not kill them. One juvenile male might have transferred to another group. Two other juvenile males remained in the range of K group. One died, and another survived at least until 1992 (Uehara et al. 1994). After Kamemanfu finally disappeared in 1982, Masisa was the only sexually mature male left. On November 26th, 1982, I happened to see Masisa. He had lost weight and looked unhealthy. He appeared concerned whenever he heard long distance calls from M group members. On December 16th, I saw him again. Masisa rushed toward a piece of sugar cane I threw to him but stumbled on the rough ground and fell. He may have been suffering from severe psychological stress. Unlike females, Masisa could not transfer to another group. This was the last time he was seen.

Infanticide and cannibalism by wild chimpanzees were first observed in the Budongo chimpanzees by Suzuki (1971). As with infanticide described in Hanuman langurs by Sugiyama (1965), Suzuki’s report was largely ignored and considered to be an abnormal event (Reynolds 2022). Later when infanticide was recognized as a sexually selected male reproductive strategy (Hrdy 1974), other cases of infanticide were documented at Mahale (e.g., Norikoshi 1982) and other field sites. Nevertheless, I never expected to witness such a rare episode.

**Fig. 4:**
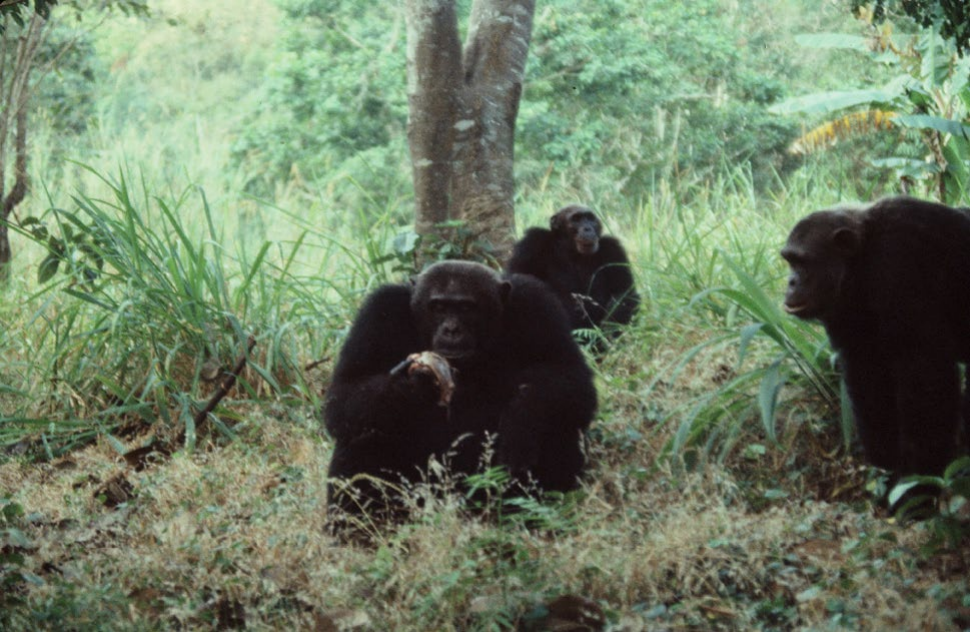
A scene of cannibalism taken hours after infanticide. Ntologi in the center, Kagimimi in the back, and Lukaja on the right. Ntologi’s right hand holds the victim. These males had joined the infanticide

On July 5th, 1983, I found three M group males attacking an adult female, Wantendele, with her newborn male infant (Takahata 1985b). The target of aggression was her son, not Wantendele. They picked up Wantendele' body to seize her male infant, who was clinging ventrally. It appeared to be a collaborative task, in which multiple individuals coordinated their activities simultaneously. Eventually, Ntologi grabbed the infant. Leaving Wantendele injured, the males began to cannibalize her infant (Fig. 4). From my observations of past mating behavior, the victim was likely to have been sired by one of the M group males. Despite these data, I speculated that this event conformed with the sexual selection hypothesis for infanticide. Wantendele had disappeared from M group for several months before giving birth, and the killers might have believed that the males from another group may have fathered the infant. On August 23rd, Wantendele resumed cycling. She mated with M group males, including Ntologi, as if both Wantendele and male killers had forgotten the infanticide. This case suggests the important role that paternity certainty plays in the lives of chimpanzees.

### Hunting and other feeding habits

From 1979 to 1981, I occasionally observed chimpanzees hunting, and Nishida advised me to summarize these data (Takahata et al. 1984). In those days, their hunting was sporadic, and their prey were juvenile ungulates or red-tailed monkeys. Sex differences existed. Male chimpanzees frequently hunted primates by chasing them, and female chimpanzees chiefly seized ungulate juveniles who froze while trying to conceal themselves.

After the 1980s, their hunting habit changed, and chimpanzees specialized in hunting red colobus monkeys (Hosaka et al. 2020). Several explanations exist for this change: improvement of observation conditions and methods, forest recovery and an increase in the red colobus population, and the invention or reinvention of new hunting habits. Whichever it is, it took 10 years for this new habit to spread.

A successful capture is followed by considerable chaos. Prey owners are disturbed by insistent begging by others, and meat consumption often lasts for several hours. Since chimpanzees' teeth and fingers were not always suitable for tearing skin and processing meat, I imagine efficient prey sharing only became possible with tools in our hominin ancestors. For early hominins like *Australopithecus*, stone tools may have been used for processing, which facilitates food sharing.

I also observed new feeding habits (Takahata et al. 1986). In the 1980s, M group began to eat the fruits of guava, mango, and lemon. These agricultural trees had been abandoned when people left their old villages following the villagization (ujamaa village) policy by the Tanzanian government in 1974. It took 7–8 years until chimpanzees began to eat these fruits. Although chimpanzees have conservative feeding habits, they are able to acquire new ones rapidly once they realize the food is edible.

### Comparison of female chimpanzee sexual behavior with bonobos

It had been pointed out that female bonobos exhibit long estrous periods and show high levels of sexual activity, but there had been no rigorous comparison of this with chimpanzees. Along with some colleagues, I analyzed chimpanzee data that had been collected at Mahale (Takahata et al. 1996). Our results revealed that female chimpanzees do not experience many estrous cycles during their lifetimes compared with bonobo females, primarily because they stop cycling while lactating. When they are in estrus, female chimpanzees actively approach and mate with mature males and immediately mate with other males. They copulate about 0.8 times per hour. Their high proceptivity facilitates promiscuous mating. From our analyses, we concluded that female chimpanzees did not always mate with the ‘best males,’ but rather with many males (but see Matsumoto-Oda 1999). Alpha males can nonetheless constrain the behavior of estrous females by monopolizing them during the period of maximum swelling. Surprisingly, the copulation frequencies of female bonobos were actually lower at 0.14–0.53 times per hour. Unlike the promiscuous mating of chimpanzees at Mahale, significant correlations existed between male bonobo rank and mating frequency. These data suggest that female bonobos exhibit ‘receptivity’ rather than proceptivity. These results were based on relatively small data sets. Male sexual coercion toward females has been frequently reported in male chimpanzees (e.g., Muller et al. 2009). In 1981 at Mahale, the frequency of coercive behaviors was low, except for Ntologi, the α male. Whether this low frequency of sexual coercion was a characteristic of the M group cannot be determined. It would repay the effort for future work to investigate this issue.

Our analyses also suggested that female chimpanzees did not synchronize their estrous cycles and ovulation periods (Takahata et al. 1996). Later, Matsumoto-Oda et al. (2007) analyzed a larger data set of observations on the Mahale chimpanzees, and also concluded that female chimpanzees did not synchronize their estrous periods. Estrus asynchrony may decrease female–female competition for mates.

## Yakushima macaques

### Wild groups on the western coast of Yakushima Islet

In October 1984, I returned to the Laboratory of Human Evolution Studies (LHES), Kyoto University, which had separated from the LPA in July 1981 when Itani became Professor. Ichikawa was appointed a lecturer in the lab. In April 1986, LHES was reorganized, and Itani and Ichikawa transferred to the Center for African Area Studies (CAAS) at Kyoto University. In those days, Itani devoted his time to establishing a research center for comprehensive regional studies in Africa. In November 1986, I became an Assistant Professor in the LHES, and Nishida was appointed the new Professor in April 1988.

In 1985, I visited the west coast of Yakushima Islet, Japan. Yakushima macaques (*Macaca fuscata yakui*) lived an uninhabited area covered by warm temperate forest. In the 1970s, when Tamaki Maruhashi (1981) started his research in this area as a graduate student at PRI, the population density was about 30 monkeys/km^2^. The mean group size was 28.8 monkeys, which was smaller than the groups in other populations inhabiting the mainland of Japan. The SSR was 0.86, suggesting that most males stayed in groups during the non-mating season.

**Fig. 5:**
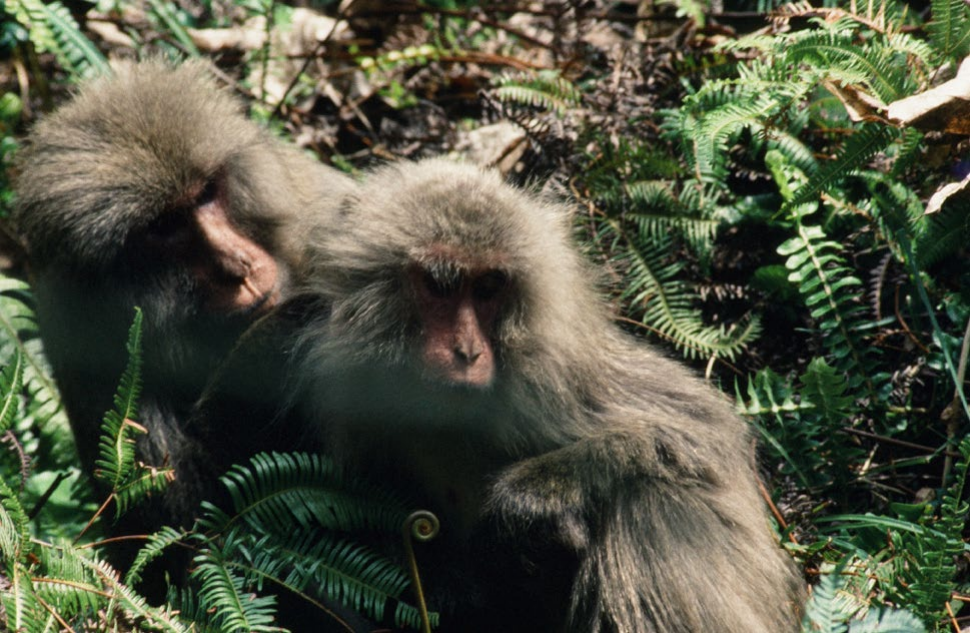
Yakushima macaques. Mars (adult male) grooms Ako (adult female) in November 1988

In 1974, Maruhashi (1982) habituated a group named Kojiba (Ko). In 1977, the Ko group consisted of 47 monkeys, but it fissioned into M (16 monkeys) and N groups (32 monkeys). Subsequently, N group fissioned again into A (31 monkeys) and H groups (11 monkeys). In 1985, two students of LHES, David Sprague (1991) and Naobi Okayasu (1992), observed the N and M groups. I took turns with Okayasu to observe M group (Fig. 5). This group consisted of 21 monkeys. No one anticipated it would disappear in 4 years.

I had many questions, including how did non-provisioned groups differ from provisioned ones? There was a significant difference in group size, e.g., 200–300 members in the Arashiyama group vs. about 30 members in Yakushima. Yakushima macaques spent much time feeding and moving, and intra-group conflicts were not conspicuous (Maruhashi 1981). Most sisters did not follow the youngest ascendency rule (Hill and Okayasu 1995). Adult males often stayed close to each other and exchanged grooming. Nonetheless, I recognized that social relations within a group were not significantly different from those of Arashiyama. What impressed me the most was that even in entirely different environments, the monkeys coexisted. They did so by coordinating their intra-group relationships and maintaining a stable rank order. Aspects of their social structure appeared to be robust.

By contrast, I observed severe intergroup competition. This might have been related to the increase in population density of monkeys in the study area. In the early 1990s, we estimated a density of 62.4–99.8 monkeys per km^2^, which was two to three times the number reported in the 1970s. We have no explanation for the cause of the increase in population density, but with the increase, intergroup relationships changed dramatically. In the 1970s, the home range of the group was regarded as a territory with little overlap with neighboring groups (Maruhashi 1982). However, in the 1980s, the home ranges overlapped considerably and aggressive intergroup encounters occurred frequently. During such encounters, smaller groups were at a disadvantage in competition with larger groups (Sugiura et al. 2000).

Around 1987, M group abruptly declined in size to just 11 monkeys, mainly because the birth rate dropped and infant mortality increased (Takahata et al. 1994). The number of adult females also began to decrease. As M group declined, males transferred to other groups one after another, the reverse of what took place with K group female chimpanzees at Mahale. Consequently, the home range of M group further decreased. By summer 1989, only one adult male, an adult female, and her daughter remained (Takahata 1991b). When the mating season started, the last male left M group, and the remaining mother and daughter finally joined a neighboring group. This was the first observed case of group extinction/fusion of Japanese macaques, and it was my second time to witness the disappearance of a primate group after the K group of Mahale chimpanzees. Researchers, including myself, thought this case was an anomaly. However, similar group extinctions/fusions have occurred since then (Sugiura et al. 2002). The coast of Yakushima is one of the richest environments for wild Japanese macaques. Nevertheless, severe intergroup competition exists. As stated above, the study population had increased rapidly since the 1970s, resulting in high densities in the 1990s. Therefore, density-dependent processes may have occurred, thereby reducing female fertility.

### Comparison between the Kinkazan and Yakushima populations

In the 1990s, a project was planned to compare the socioecological features between the Yakushima and Kinkazan populations (Yamagiwa et al. 1998). These populations live in different environments. On the western coast of Yakushima, the mean temperature was 20 ℃, annual rainfall was about 3000 mm, and the broad-leaved evergreen forest grew. At Kinkazan, vegetated by broad-leaved deciduous temperate forests, the mean annual temperature was 11 ℃ and the annual rainfall was about 1500 mm. Neither site had predators. We examined how these different environments affect their social lives.

One component of this study was to investigate female reproduction (Takahata et al. 1998a). The population density was much higher in the coastal forest of Yakushima (62.4–99.8 monkeys/km^2^) than in Kinkazan (25.0–28.0 monkeys/km^2^) due to differences in fruit production. However, the birth rate at Yakushima was lower than that at Kinkazan. One explanation for these results is that Yakushima females compete more severely for resources. Alternatively, the population density at Kinkazan might be limited by climatic factors, such as heavy snow, rather than density-dependent ecological effects.

We also analyzed the relationship between group size/number of adult females of each group and reproductive parameters (Takahata et al. 1998b). At Yakushima, where group size was small, the birth rate increased with group size / number of adult females because a larger group may gain an advantage in intergroup competition. By contrast, the birth rate is likely to decrease with the number of adult females at Kinkazan, where the group size was large, because intra-group competition may increase in larger groups. Thus, our data are consistent with Wrangham’s (1980) model of female-bonded primates.

### Area surveys and vertical distribution of Yakushima macaques

In 1991, Kagoshima Prefecture requested a census of monkey populations around the coastal area of Yakushima Islet, mainly in areas where monkeys were raiding farm crops. Since the 1970s, crop raiding by monkeys had been increasing. This, in turn, was due to two factors: the deforestation of natural forest and the decline of villages due to Japanese agriculture and economic policies.

Traditional census methods, such as line transects, were difficult to conduct because of steep mountains and dense vegetation. Instead, I modified the “fixed observers in quadrats (FOIQ)” method devised by Oi et al. (1994) by dividing the research area into 500 m × 500 m quadrats, with observers positioned at a fixed point in each quadrat. Four observers participated in one party covering an area of 1 km^2^ with one leader. When observers heard or saw monkeys, they informed the leader by radio, and the leader counted the number of monkeys.

A large team was led by Shinichi Yoshihiro (Associate Professor of Ryukoku University). Most participants were students from various schools and universities, and it was necessary to train them. I taught them how to make a weather chart by listening to the radio broadcast (in the 1990s, weather charts were not available on the internet). In our census area of 127.3 km^2^, 131 groups and 2000–3850 monkeys were estimated to inhabit the forest (Yoshihiro et al. 1998). Monkeys were dependent on natural broad-leaf forests, and group density was highest in the western coastal area of Yakushima.

From 1994 to 1997, we applied the FOIQ method to investigate the vertical distribution of monkeys (Yoshihiro et al. 1999). In the coastal forest (< 300 m above sea level.) 4.8 groups and 62.4–99.8 monkeys per km^2^ were estimated to inhabit the area. The estimated values abruptly decreased to 1.3–1.6 groups/km^2^ and 30–36 monkeys/km^2^ in the mountain slope forests (300–900 m a.s.l.). About 2.4 groups/km^2^ and 36 monkeys/km^2^ lived higher in the coniferous forests (> 900 m a.s.l.). In the conifer forest zone, monkeys relied on fiber-rich foods (e.g., mature leaves). Such ecological differences likely influenced their population density (Hanya 2004).

**Fig. 6:**
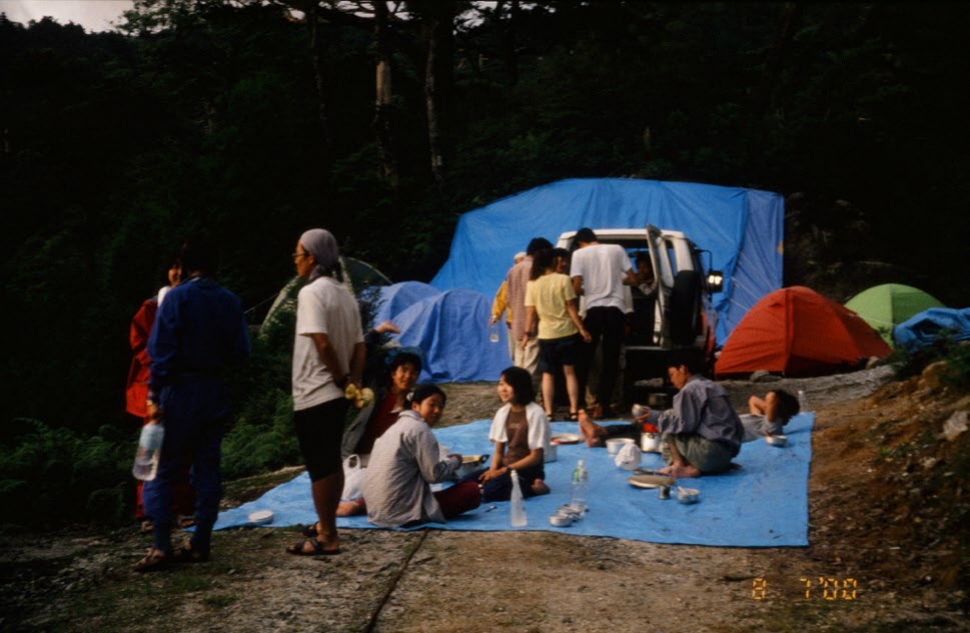
Participants at the campsite in the evening

The preceding studies indicate the value of Yakushima as a field site for investigating how environmental factors influence the socioecology of Japanese macaques. For example, ecological conditions impacted intergroup competition, while social relationships within the group were less affected (Hanya et al. 2008). In coniferous forests, intergroup competition did not exist and differed from what occurred in the coastal forest. Consistent differences did not exist in the frequency of intra-group agonistic interactions and female social relationships in the coniferous and coastal forests. Goro Hanya (Associate Professor of The Center for Ecological Research, Kyoto University) and his colleagues continue this census project today, and many student participants have gone on to become scientists, including primatologists (Fig. 6).

## Ringtailed lemurs at Berenty, Madagascar

### Berenty reserve

I left Kyoto University, transferring to the Naruto University of Education in 1992 and Kwansei Gakuin University in 1996. Due to workplace circumstances, I had few opportunities to conduct long-term fieldwork. On the other hand, I made use of my experience as a primatologist to provide academic support to students with various disabilities at Kwansei Gakuin University. I investigated what prevented them from understanding lessons in class, and I devised measures to help them and set up support systems. This included computer-based note-taking for hearing-impaired students and the production of materials in Braille for visually impaired students.

In 1997–1998, I had a chance to observe wild ringtailed lemurs (*Lemur catta*) for four months as a member of project organized by Naoki Koyama (Professor at CAAS), together with his students, Naomi Miyamoto and Shinichiro Ichino. Understanding the evolution of human society requires the study of all members of the Order, including strepsirrhines, monkeys, and apes.

At the Berenty Reserve, Madagascar, a population of ringtailed lemurs had been observed by Alison Jolly and her colleagues since 1963 (Jolly et al. 2006). The reserve is surrounded by the Mandrate River and a sisal plantation. Plantation workers reminded me of the rubber plantation workers in Sumatra. Annual precipitation was about 580 mm, and 70% of the rainfall fell between November and February. As one moved away from the river, the vegetation drastically changed from gallery forest dominated by *Tamarindus indica*—a staple food for lemurs—to scrub forest and semi-desert. This reminded me of environmental changes from the coast to mountain summits of Yakushima. Depending on this gradient, the population densities of lemurs also changed.

The ringtailed lemur is a strict seasonal breeder, with a birth peak in September (Koyama et al. 2001). The annual birth rate of adult females was 75–85%. The mean lifespan of females was 4.9 years, with the longest being 20 years. Old-aged females continued to give birth without PRLS. Infant mortality was as high as 37.7% (Ichino et al. 2015). Long-term environmental changes influenced the reproduction of females. In the 2000s, tamarind trees began to decline. In addition, the Reserve office stopped the feeding by tourists and water supply. Consequently, lemur body mass decreased from 2.2 to 2.3 kg in 1999 (Koyama et al. 2008) to about 2.0 kg in 2006 (Ichino et al. 2013). Interestingly, females maintained a high birth rate (73.0%), but infant mortality became high (86.2%). In the unstable environment of arid forests, ringtailed lemurs may have evolved an r-strategy for reproduction.

### Social relationships among ringtailed lemurs

In 1989, Koyama set up a study area of 14.2 ha in the center of the Reserve and started a long-term project (Koyama et al. 2006). Ringtailed lemurs form female-bonded/matrilineal multi-male and multi-female groups of 10–30 individuals. They exhibit female philopatry. Males leave their natal groups when they are around 3 years old and move to non-natal groups with a tenure length varying from 1 to 7 years. This social life resembles Japanese macaques, although there are many differences too. Such differences can tell us something about the evolution of matrilineal social structures.

The ringtailed groups were much more unstable than those of Yakushima macaques (Koyama et al. 2002). In September 1989, three groups lived in our study area. However, during 10 years, eight group fissions, six evictions of females, and three range takeovers by other groups occurred. Female evictions proceed as follows. In a group, one or several females became the target of persistent aggression from other females, lasting several days or weeks. This targeted aggression eventually led to the eviction of the targeted females from their group. If mature males joined these females, and they could secure a home range, the process led to group fission. However, in many cases, the evicted females could not secure ranges and eventually disappeared from our study area altogether.

**Fig. 7:**
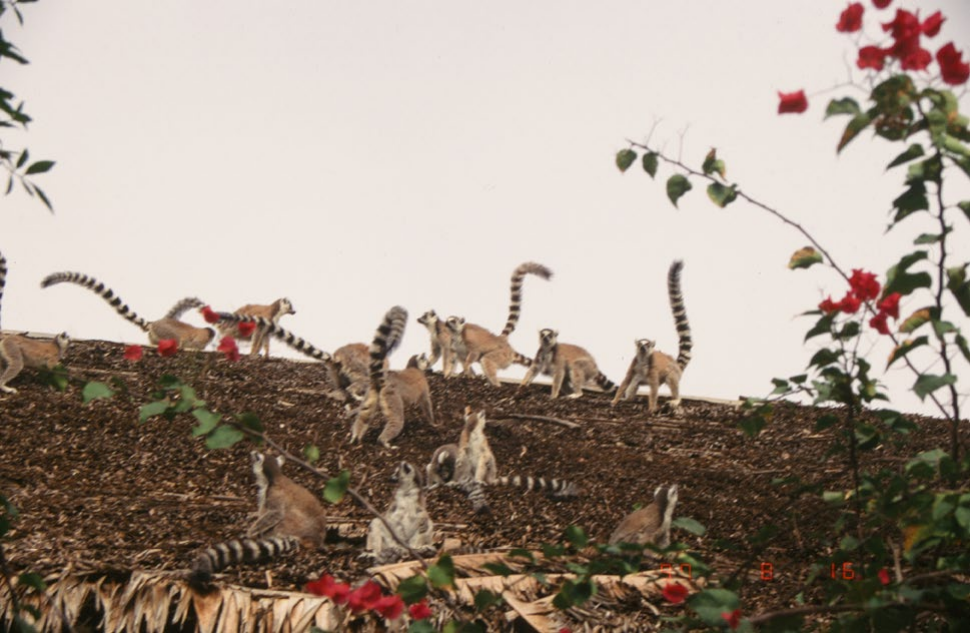
Encounter between the C1 and C2 lemur groups on the roof of a restaurant for tourists. Females were at the forefront of these conflicts

All cases of group fission and female eviction occurred in relatively large groups of >20 lemurs, suggesting severe intra-group competition (Takahata et al. 2006). When groups encountered each other, smaller ones tended to be overwhelmed by the larger one, suggesting that smaller groups of females were at a competitive disadvantage (Fig. 7). Females in small and large groups showed lower birth rates compared with females in mid-sized groups, similar to the cases reported in Japanese macaques. In this respect, targeted aggression might be regarded as a way for females to maintain optimal group size.

Each group had a linear rank order involving males and females (Koyama et al. 2005). Female dominance over males was a prominent feature. There is considerable debate about why females are socially dominant to males in lemurs (Jolly 1984), and I do not have any convincing answers. Female dominance intrigued me because until then I had been studying primates in which males were the dominant sex. One day, an adult male was drinking water. In the drylands of Berenty, the order of drinking water is a clear indicator of rank. He suddenly noticed a 2-year-old adolescent female approaching. He immediately withdrew, giving access to the water source to her. In this case, female dominance worked through male deference.

Female rank order fluctuated. Females maintained their alpha status about 2 years on average. Unrelated females constantly competed for rank. Once, I observed direct female competition unexpectedly (Takahata et al. 2001). In 1997, a female gave birth during the day. After that, an unrelated subordinate female kicked her hard, and outranked her after that. Ringtailed females occasionally give birth during the day. Out of the 16 daytime deliveries, attacks on new mothers occurred twice.

Among kin-related females, mothers were usually dominant over their daughters. Older sisters were habitually dominant over younger sisters (i.e., the youngest ascendency did not exist). Young females usually occupied the lowest ranks among adult females, but several of them attained higher ranks right beneath their high-ranking mothers, indicating the existence of “dependent ranks.” In these situations, young females relied on the influence of their mothers to acquire higher ranking positions over older females.

**Fig. 8:**
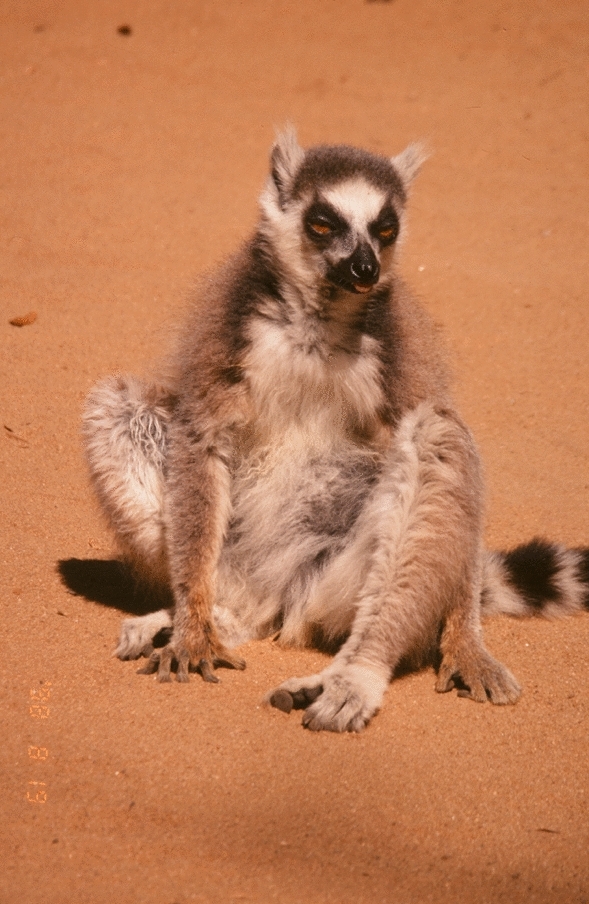
KI, an old female ringtailed in the C1 group

Do ringtailed lemurs have memories of past social relations? One day, I observed the Cx group. Jolly suddenly asked me from behind. “Who is this female”? I looked back, and KI, the oldest female in the C1 group, was sitting there (Fig. 8). Jolly had followed her all the way here. KI was very old and frequently got separated from her group and wandered alone in the range of other groups due to her senility. I replied, “She is a C1 group female named KI. She is wandering out of her group’s range.” Then, I noticed that KI-92 (KI’s son, who had left C1 group 3 years ago) was 5–6 m away from her. However, there was no particular interaction between the mother and son, and KI slowly moved away. Therefore, it is uncertain whether they recognized each other or not.

## Summary and hope for the future

As stated above, I have adopted an inductive approach and searched for topics to study at each field site where I was given a chance to work. This is not the way most current primatology operates, prioritizing theory over descriptive study. As Shirakami taught us, theories are necessary to make our discoveries important. When I started my research career, paradigmatic changes were taking place, and I did not always receive guidance regarding the revolution that was occurring in behavioral ecology. Once, at a seminar, my presentation about the reproductive biology of the Arashiyama B group received a severe comment that “your topic is not at all within the category of animal ethology.” Nonetheless, the paper that resulted from that presentation is one of my most frequently cited studies. In retrospect, if I had received different advice about the changes in thinking that were occurring regarding female competition and female life history, my doctoral thesis might have become a little more “important."

Looking back, Japanese primatology relied on Imanishi and Itani for theory in 1950s to the 1970s. Most of those who followed them, including me, tended to devote ourselves to empirical field research. What we need now is to build new theories from observations based on our fieldwork. In 1993, I made a presentation at PRI entitled “Only yesterday: reviewing the Japanese primatology of the 1980s and the future.” I stressed the importance of using our discoveries to create new theories (Takahata 1994). Unfortunately, this has not always taken place. I hope young scholars will learn how to make their discoveries meaningful by combining theory with their field observations.

During my research career, I have always been interested in the origins of the human family. The cases of PPRs at Arashiyama furnish a faint approximation of the human family. The lack of mating relationships between pairs, however, do not make them an especially strong analogy. I have also turned my interest to the reproductive life history of female chimpanzees. At Mahale, the interbirth interval of females is about 6 years (Nishida et al. 1990). This limits their lifetime reproductive success. This is consistent with mammalian life history where parenting is left to females. At some point in human evolution, human females gained direct or indirect support from long-term relationships with particular males to help them parent and increase their fertility. As a result, a social system called the ‘family’ may have emerged where pairs of females and males raise multiple children who are dependent on their parents (Lancaster 1991). I hope that research on female choice and/or female competition will make further progress and that it will contribute to elucidating the evolution of human society. At the same time, I hope research on various aspects of sexuality will progress, including genetics, physiology, and behavior.

## Data Availability

The datasets analyzed in this study are available from the corresponding author on reasonable requests.
